# Analysis of fluoroquinolones in dusts from intensive livestock farming and the co-occurrence of fluoroquinolone-resistant *Escherichia coli*

**DOI:** 10.1038/s41598-019-41528-z

**Published:** 2019-03-26

**Authors:** Jochen Schulz, Nicole Kemper, Joerg Hartung, Franziska Janusch, Siegrun A. I. Mohring, Gerd Hamscher

**Affiliations:** 1 Institute for Animal Hygiene, Animal Welfare and Farm Animal Behaviour, University of Veterinary Medicine Hannover, Foundation, Hannover, Germany; 2 Institute of Food Chemistry and Food Biotechnology, Justus Liebig University Giessen, Giessen, Germany; 3 Present Address: Eurofins WEJ Contaminants GmbH, Hamburg, Germany; 4 Present Address: Thermo Fisher Scientific GmbH, Bremen, Germany

## Abstract

Fluoroquinolones are important therapeutics in human and veterinary medicine. This study aimed to retrospectively analyse sedimentation dusts from intensive-livestock-farming barns for fluoroquinolones and investigate the association between resistant *Escherichia coli* and the detected drugs. Sedimentation-dust samples (n = 125) collected (1980–2009) at 14 barns of unknown-treatment status were analysed by HPLC and tandem-mass spectroscopy to detect enrofloxacin, ciprofloxacin, marbofloxacin, and difloxacin. Recent microbiological data were included to investigate the relationship between fluoroquinolone presence and fluoroquinolone-resistant *E. coli*. Fifty-nine dust samples (47%) from seven barns contained fluoroquinolone residues. Up to three different fluoroquinolones were detected in pig and broiler barns. Fluoroquinolone concentrations ranged from 10-pg/mg to 46-ng/mg dust. Fluoroquinolone-resistant *E. coli* were isolated from four barns. Of all the dust samples, 22% contained non-susceptible isolates. Non-susceptible isolate presence in the dust was significantly associated (p = 0.0283) with detecting the drugs, while drug detection increased the odds (4-fold) of finding non-susceptible *E. coli* (odds ratio = 3.9877, 95% CI: 1.2854–12.3712). This retrospective study shows that fluoroquinolone usage leads to dust contamination. We conclude that farmers and animals inhale/swallow fluoroquinolones and fluoroquinolone-resistant bacteria due to drug application. Furthermore, uncontrolled drug emissions via air exhausted from the barns can be assumed.

## Introduction

Fluoroquinolones are important therapeutics used to treat human and animal infection. For instance, the introduction of fluoroquinolones offered clinicians the ability to treat human cases of complicated urinary tract infections, gastrointestinal infections, sexually transmitted diseases, and respiratory tract infections^1^. The first fluoroquinolones were introduced into human medicine in 1984^2^. In veterinary medicine, fluoroquinolones have been effective therapeutics for treating enteric infections and respiratory diseases in food-producing and companion animals^3^. Their antimicrobial activity against a broad spectrum of pathogenic bacteria, advantageous pharmacokinetic characteristics, and low toxicity make them attractive for use in farmed animals^4,5^. Since the late 1980s, the fluoroquinolones used in human medicine have differed from the compounds used in veterinary medicine^6,7^. However, a public health concern is that the use of fluoroquinolones in livestock selects for bacterial resistance that can be transmitted into the food chain. For instance, the transmission of ciprofloxacin-resistant *Salmonella* and *Campylobacter* spp. from food animals to humans has been suggested, even though ciprofloxacin is not used in animal husbandry^6,7^. This can be explained by the cross-resistance between fluoroquinolones and because enrofloxacin, a commonly used agent in farm animals, is partially metabolized to ciprofloxacin in animals^8,9^.

Livestock receive intensive antibiotic treatment through their drinking water and feed. Furthermore, a portion of the antibiotic is excreted from the animals unaltered^10^. Thus, there are three potential sources for fluoroquinolones in animal husbandries that could lead to the contamination of the excrement, litter, surfaces, and air. For example, it is known that feed and excrement particles are part of the airborne dust in animal houses^11^, which could contribute to the undesirable dissemination of antibiotics within them. Hamscher *et al*.^12^ detected different antibiotics, including various sulfonamides, tetracyclines, and tylosin, in the sedimentation dust from a pig barn, even after two decades of storage. The dust that settled 1.5 m above ground was previously airborne, meaning it was potentially inhalable by people and animals in the barn. The fluoroquinolone content was not analysed in the study by Hamscher *et al*. in 2003 nor by McEachran *et al*.^13^ in a more recent study, raising the question of whether the dust from these farm-animal houses might also contain these drugs.

Furthermore, Scherz *et al*.^10^ recently showed that the application of enrofloxacin induced the development of resistant commensal *Escherichia coli* in the intestines of poultry. Commensal *E. coli* can be excreted by farm animals, enabling its detection in farm-animal dust, even in more than 20-year-old samples^14^. The possibility of detecting antibiotics and antibiotic-resistant bacteria in stored sedimentation dust samples may provide an opportunity to obtain information retrospectively about the co-occurrence of fluoroquinolones and fluoroquinolone-resistant bacteria. Given these circumstances, important questions arise regarding the use fluoroquinolones in the past, their concentrations in the dust, and the exposure of the farmers, animals, and environment to the airborne dust. Thus, in this study, we analysed 125 dust samples collected in different animal husbandries from 1980 to 2009 and compared their residue data with the corresponding results of a recently conducted microbiological study^14^.

## Results

### Detection of fluoroquinolones

Our modified sample extraction method combined with the recently established high performance liquid chromatography electrospray ionization tandem-mass spectrometry (HPLC-ESI-MS/MS) method permitted the selective and sensitive detection of various fluoroquinolones in the dust samples. The validation parameters listed in Table S2 demonstrate that the method is well suited for investigating dust samples for the four fluoroquinolones at a broad range of concentrations. One should keep in mind that the minimum inhibitory concentration (MIC_50_) values of the fluoroquinolones under investigation ranged from 0.002 to 0.25 mg/L in a broad panel of recently isolated porcine and bovine bacterial pathogens. Comparing these values to the limit of detection (LOD) of our current HPLC-ESI-MS/MS method (0.005 µg/kg), we detected antibiotic concentrations at least 400-fold lower than the lowest MIC_50_ value obtained for *Pasteurella multocida*^4^. Furthermore, the superb performance of the analytical procedure was achieved with a very low sample amount of only 50 mg.

**Figure 1:**
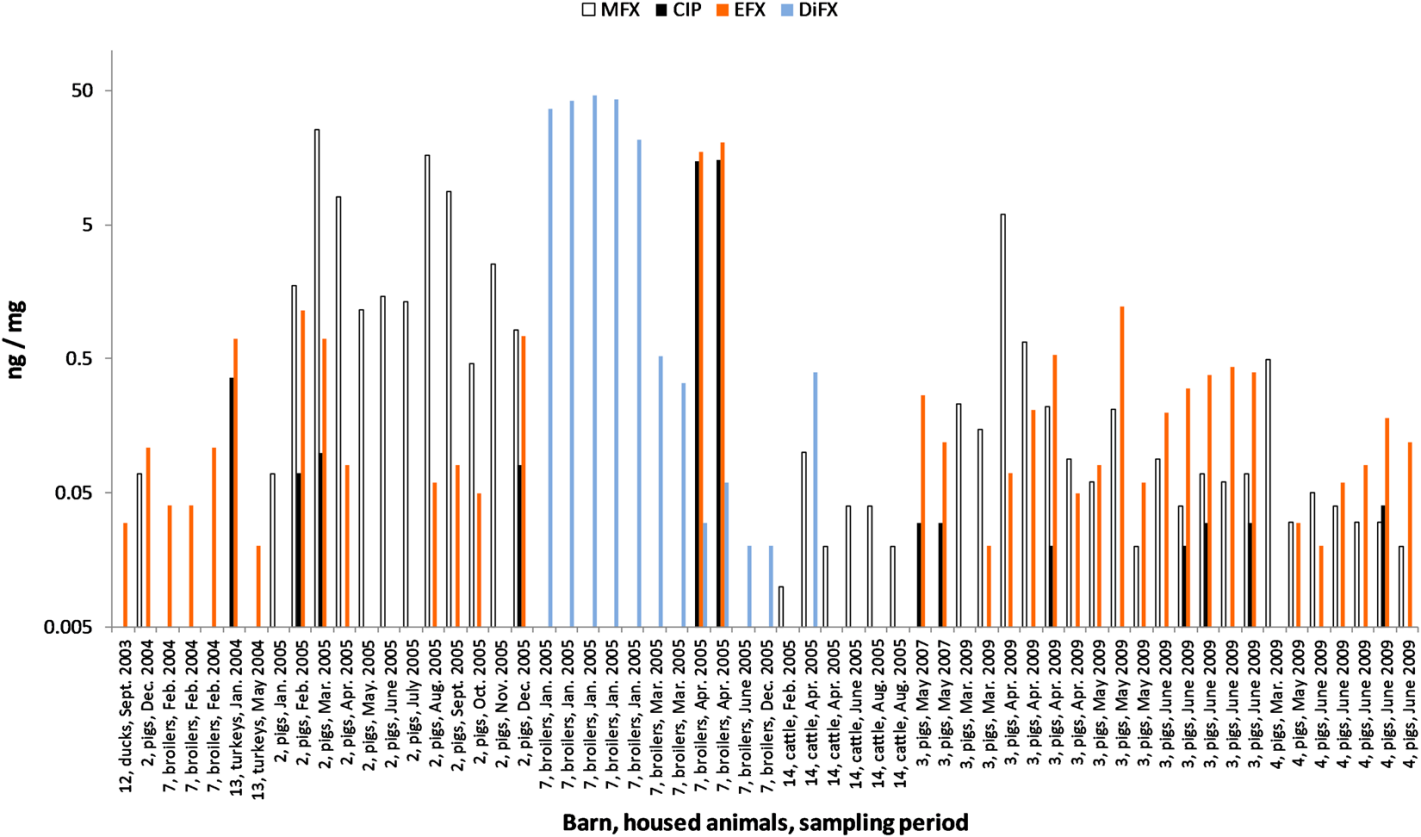
Concentrations of marbofloxacin (MFX), ciprofloxacin (CIP), enrofloxacin (EFX), and difloxacin (DiFX) in the sedimentation dusts from Barns 2, 3, 4, 7, 12, 13, and 14.

Various fluoroquinolones were detected in 59 (47%) of the 125 dust samples, and positive samples originated from 7 (three pig barns, three poultry barns, and one cattle barn) of the 14 sampled barns. Fluoroquinolones were found in a wide range of concentrations, from 0.01 ng/mg up to 46 ng/mg (see Fig. 1). The earliest detection was from a sample acquired in 2003. The samples from the 1980s and 1990s were below the limit of detection (LOD). Two or more fluoroquinolones were detected in six of the barns. Marbofloxacin was found in 40 dust samples from the pig barns and one cattle barn, but the dusts from the poultry barns were consistently below the LOD. In 27 cases, marbofloxacin was detected together with enrofloxacin and/or ciprofloxacin. In four barns (Barns 2, 3 and 4 from 2009 and Barn 14 from 2005), marbofloxacin was present in every sample for all the sampling periods, indicating continuous usage. Ciprofloxacin was only found in the dust from pig and poultry barns when enrofloxacin was present. If both antibiotics were detected simultaneously, the enrofloxacin was always found in higher concentrations. The concentrations of the two antibiotics were not normally distributed (Kolmogorov-Smirnov test, *p* < 0.0100), and a signed-rank test revealed significant differences between the concentrations (*p* = 0.0002). However, no ciprofloxacin was found above the LOD in 65% of the enrofloxacin-positive samples (n = 24). Difloxacin was detected at high concentrations (up to 46 ng/mg) in the dust from a broiler barn (Barn 7) and in one sample (0.4 ng/mg) from the cattle barn.

### Association between the presence of fluoroquinolones and non-susceptible *E. coli* in the dust

*Escherichia coli* were detected in 54 (43%) of the analysed dust samples. In 28 cases (22%), non-susceptible *E. coli* were also isolated. The non-susceptible isolates were assigned to four different phylogenetic groups (A, B1, E, and D), and different phylogenetic groups were found in Barns 2 (A, B1) and 7 (B1, E, D) (see Table S1). To analyse the associations between the presence of isolates capable of growing on ciprofloxacin-containing media (non-susceptible isolates) and the detection of fluoroquinolones, a contingency table (Table 1) was generated from the results in Table S1. A Fisher exact test showed a significant association between the presence of *E. coli* with reduced ciprofloxacin susceptibility and the detection of fluoroquinolones in the same dust sample (*p* = 0.0283). The odds ratio was calculated to estimate the chances of finding potential fluoroquinolone-resistant *E. coli* in samples with fluoroquinolone residues. The result indicated that the odds of finding *E. coli* with reduced susceptibility to ciprofloxacin was approximately four times higher (odds ratio = 3.9877, 95% CI: 1.2854–12.3712) in the dust samples with fluoroquinolones versus those without.

**Table 1:** Growth of *Escherichia coli* with reduced ciprofloxacin susceptibility from dust samples with and without detected fluoroquinolones.

Growing on Medium	Fluoroquinolones in the Dust Sample	
	Yes	No
With ciprofloxacin	19	9
Without ciprofloxacin	9	17

## Discussion

The dust samples analysed in this study were originally sampled within the scope of other investigations and thus represent heterogenic material. However, all samples were stored under the same conditions, which allows for comparable analyses^14^. Unfortunately, no information was available regarding antibiotic treatments in the corresponding barns. To determine if the farm animals were treated with fluoroquinolones and to what extent their residues could be detected in the dust, three drugs (marbofloxacin, enrofloxacin, and difloxacin) approved for use in veterinary medicine in Europe were chosen for the analyses^15,16^. Ciprofloxacin was included because it represents the most important metabolite of enrofloxacin in farm animals^17^. Furthermore, it is recognized as a critically important antimicrobial in human medicine^18^. Fluoroquinolones were introduced for treating food animals in Europe in the late 1980s^19^. In 48 samples collected from four different barns between the 1980s and 2000, no fluoroquinolones were found above the LOD. Negative results for the samples from the 1980s might be expected, and the uncontaminated dust of later samples may reflect a restricted use of these antibiotics. It is well known that fluoroquinolones are synthetic compounds with a high stability in various biological and environmental matrices. This results in poor metabolization, e.g. by mammals, and large amounts of the drugs are excreted unchanged^20^. Photodegradation of fluoroquinolones has been primarily demonstrated in aqueous media. However, in solid materials such as soil, the fluoroquinolones bind strongly to the material, and their biodegradation is markedly reduced^21^. Thus, any relevant degradation of the fluoroquinolones in the barns or dry sedimentation dust samples is in combination with our controlled storage conditions highly unlikely.

Fluoroquinolones were first detected in a duck flock in 2003 and subsequently, in barns occupied by broilers, pigs, and cattle. This may suggest wide usage of the drugs, but one should keep in mind that this is not a representative study regarding the sampling strategy and number of barns. Nevertheless, to the best of our knowledge, this is the first study to demonstrate persistent fluoroquinolone residues in dusts from different farm-animal houses after several years of storage. Although it is not known what influence the storage had on the concentrations, the methods used allowed for a look into the past and may show that farmers were using fluoroquinolones more than a decade ago. The individual treatment methods in our retrospective study are unknown, but fluoroquinolones are administered via drinking water or injections, and this treatment of the herd can result in particles from the litter and faeces as well as droplets of drinking water containing the drugs^10^. These particles and droplets can become airborne, with their ingredients becoming a part of the sedimentation dust^12^.

In this study, up to three different fluoroquinolones were detected in a single dust sample. Ciprofloxacin is a metabolite, but the simultaneous detection of enrofloxacin and difloxacin in the dusts from Broiler Barn 7 and of enrofloxacin and marbofloxacin in the dusts from Pig Barns 2, 3 and 4 suggests the animals were treated with different fluoroquinolones. Day-old chicks, for instance, are not treated when they arrive at broiler barns. Thus, the simultaneous detection of enrofloxacin and difloxacin in samples from Broiler Barn 7 indicates that different antibiotics were administered in this barn. However, the concentrations of difloxacin measured in April, June, and December 2005 were relatively low and might be the result of carry-over from prior growing cycles. In the case of the pig barns, we cannot exclude that the pigs were treated prior to arriving at the barns, because pigs excrete detectable amounts of enrofloxacin as much as six days after treatment^22^. Although it seems clear that recently treated animals introduced into a different barn can be a source of antibiotics, we were not able to estimate if this route contributed to the detectable amounts in the sedimentation dusts.

Overall, the concentrations of the fluoroquinolones in the dusts from different husbandries varied over a broad range of concentrations (more than three logs). All of the antibiotics analysed in this study reached maximum concentrations greater than 10 ng/mg dust. This is in good accordance with the results of Hamscher *et al*.^12^, who found various antibiotic concentrations in stable dust ranging from 0.23 to 12.5 mg/kg (=ng/mg). Taking both studies together, surprisingly high concentrations of these anthropogenic compounds were found in the environmental matrix. This is also true for ciprofloxacin, but at significantly lower concentrations than the enrofloxacin, which supports our assumption that the ciprofloxacin is present as a metabolite of the parent drug.

Dust contamination can be influenced by many factors such as the dosage and drug formulation^23^, other ingredients in the dust, farm hygiene, and the housing system. Considering the average amounts of inhalable dust in pig and poultry barns of approximately 2 and 4 mg/m^3^, respectively^24^, it can be assumed based on the maximum fluoroquinolone levels in the dusts and on a minute volume of 6.8 l/min^25^ that farmers could inhale 0.17 µg and 0.61 µg per day, respectively, when working eight hours in the barns. Considering the minute volumes from Fedde *et al*.^26^ and Reinhold *et al*.^27^, broilers (2 kg) and pigs (100 kg) could inhale 0.39 µg and 2.45 µg per day, respectively. These inhaled amounts are far below the therapeutic dosages for humans and farm animals^10,28,29^. This means the farm workers and animals inhale and probably swallow sub-therapeutic concentrations of fluoroquinolones, which could influence the resistant microbiota of the individuals^10^.

Another concern is that barn emissions contaminate the air, soil surfaces, water, and plants in the vicinity of the buildings, and this might impact the microbiomes in these environments^30–32^. For example, using the average emission factor for dust emitted from fattening pig units with 1000 pigs weighing 100 kg each, 135.6 g/h are released into the outer air^30^. Given that 1 mg of dust in a pig barn can contain 10 ng of fluoroquinolones, it can be assumed that when pigs are treated, approximately 1.4 mg of the drugs are emitted from the barn per hour. Such emissions can probably be reduced with the installation of air-filtration systems (end of pipe). However, the reduction efficiency for airborne antibiotics is thus far unknown, and these systems are only used in rare cases.

There is no doubt that the use of antibiotics in animal husbandries causes the dispersion of antibiotic-resistant bacteria^33^. Treating chickens with enrofloxacin, for instance, increases the shedding of fluoroquinolone-resistant *E. coli*.^10^ Huang *et al*.^34^ and Pourcher *et al*.^35^ measured 10^10^ CFU/g and 1.9 × 10^5^ CFU/g of fluoroquinolone-resistant *E. coli* in faecal samples from treated pigs and in manure from treated chickens, respectively. Schulz *et al*.^14^ detected resistant *E. coli* in stored sedimentation dust from pig and poultry barns in much lower concentrations (3.1 × 10^2^ to 3.9 × 10^4^ CFU/g, median = 2.3 × 10^3^ CFU/g). Taking the median concentration in dust and the assumptions mentioned above into consideration, pigs could inhale approx. 2 × 10^2^ CFU, whereas farmers and broilers would inhale approx. 2 × 10^1^ CFU airborne fluoroquinolone-resistant *E. coli* per day. The authors discussed the presence of ciprofloxacin-resistant *E. coli* in the dust carefully because the treatment states of the barns were unknown, and untreated animals could also have shed the ciprofloxacin-resistant *E. coli*. Thus, the chemical analysis of the same dust samples that Schulz *et al*.^14^ used led to our investigation of an association between the presence of fluoroquinolones and non-susceptible *E. coli*.

Schulz *et al*.^14^ regarded *E. coli* as non-susceptible when the bacteria were able to grow on ciprofloxacin-supplemented media. The MIC values for the isolates growing on these media are shown in Table S1. Based on the breakpoints published by the Clinical and Laboratory Standard Institute (CLSI 2014), 100% of the tested isolates were non-susceptible and 96% can be regarded as resistant. Although the total number of samples showing the co-occurrence of non-susceptible *E. coli* and fluoroquinolones was limited, the results were significant. In other words, the detection of fluoroquinolones in animal husbandries increased the chances of finding non-susceptible *E. coli* in the dust. However, this result is from basic statistics, because important predictors (examples given in further discussion) are missing to, for instance, perform logistic regression models. Nevertheless, showing that the antibiotics and *E. coli* non-susceptible to ciprofloxacin occur together was probably possible for two reasons. First, resistance to one fluoroquinolone decreases susceptibility to other members in this antibiotic class^18^. Second, the fluoroquinolones that were investigated are (still) those most used to treat farm animals in Germany^36^.

The selection process that increased the fluoroquinolone-resistant *E. coli* in the dust samples probably occurred in the treated animals or in the fluoroquinolone-contaminated animal environment. In this respect, enrichment in the environment by sub-MIC selective effects should be considered^32^. However, selection for resistant bacteria in the sedimentation dust itself is highly unlikely, because the water activity is far too low for *E. coli* metabolism^14^. Resistant *E. coli* isolates in the same barns were assigned to different phylogroups. For instance, three different phylogroups were found in Barn 7 (broilers), and two were found in Barn 2 (pigs). These results suggest that different fluoroquinolone-resistant clones were introduced into the barn. However, the detection of *E. coli* and fluoroquinolones in the dust might have been affected by several unknown factors. For example, treatment status and drug administration details are important factors that very likely influenced the results^10^. Furthermore, fluoroquinolone-resistant *E. coli* were isolated from dusts from broiler barns without fluoroquinolone residues. This could have been due to the colonization of young chicks with resistant *E. coli* via contaminated eggs^10^ or to bacterial contamination that remained in the barns after cleaning and disinfection measures^37^. Otherwise, samples from the pig and poultry barns contained fluoroquinolone residues and only susceptible isolates. The number of cultivatable ciprofloxacin-resistant *E. coli* isolates versus total *E. coli* isolates is generally lower in dust samples from pig and poultry barns^14^. Assuming that faeces are the main source of *E. coli* in the dusts, the findings from Schulz *et al*.^14^ are in accordance with those from Taylor *et al*.^38^, who showed that the proportion of fluoroquinolone-resistant *E. coli* compared to total *E. coli* is always lower in poultry faeces (0.0005% to 37%) and predominately lower in pig faeces (0.008% to 53%), even though fluoroquinolones were used in a quarter and two-thirds of the sampled poultry and pig barns, respectively.

When fluoroquinolones are applied in a barn, the farmers and even the untreated animals are at risk for exposure to both the drugs and resistant bacteria. The accidental inhalation or ingestion of dust in these environments could lead to the unwanted colonization of fluoroquinolone-resistant bacteria of the farmers and animals, and consequently, to the additional dissemination of resistance^10,39^. This latter point and the fact that antibiotics and antibiotic-resistant bacteria contaminate the animal environment and barn vicinity underline the necessity of using antibiotics prudently in farm animal husbandries. In the case of treatment, farmers have the option of preventing airborne transmission to themselves by wearing respirators. The animals remain unprotected, however, and if no filtering systems are installed to clean the air exhausted from the barns, fluoroquinolones and other antibiotics are emitted uncontrolled into the environment.

This retrospective study showed first that the use of fluoroquinolones leads to an association between fluoroquinolone and fluoroquinolone-resistant *E. coli* contamination of the dust produced in intensive livestock farming. We conclude that farmers and animals inhale and swallow fluoroquinolones and fluoroquinolone-resistant bacteria due to the application of the drugs inside the barns. Furthermore, it can be assumed that uncontrolled emission of the drugs into the environment via exhaust air from the barns is occurring.

## Materials and Methods

### Dust samples analysed

One hundred and twenty-five sedimentation dust samples originating from five pig barns, eight poultry barns, and one cattle barn (all located in Northern Germany) were included in this study. The dusts were sampled and stored as described in detail by Schulz *et al*.^14^. Briefly, the dusts were collected from a defined surface 1.5 m above the barn floor and stored in sterile glass cylinders in an air-conditioned room at 4 °C in the dark. The origin of the individual samples, barn numbers, and sampling periods are provided in Table S1 of the Supplemental Materials.

### Fluoroquinolone analysis

To measure the fluoroquinolones, we employed a method already described in detail by Janusch *et al*.^15^ with minor modifications regarding the sample preparation. Briefly, we extracted 50 mg of sample with 10 mL dichloromethane for 15 min. To obtain high recoveries, the extraction procedure was repeated three times. For the unequivocal detection and quantification of the fluoroquinolones, we employed HPLC-ESI-MS/MS. The methodological and instrumentation set-up for this part of the analytical procedure was also performed as described by Janusch *et al*.^15^. Due to this being a first application of this slightly modified analytical method to a new matrix, a comprehensive method validation was performed that included important parameters such as the accuracy, intra-day precision, inter-day precision, linearity, LOD, and limit of quantification. Fluoroquinolone-free dusts from sampling years before 1990 were chosen for this investigation. All calculations for the validation parameters were performed according to Janusch *et al*.^15^ and references therein.

### Comparison with bacteriological findings

To analyse the potential for associations between the fluoroquinolones detected and the presence of fluoroquinolone-resistant bacteria, the results of previous microbiological investigations by Schulz *et al*.^14^ using most of the same dust samples were included in this study. These authors isolated *E. coli* from media with and without ciprofloxacin supplementation to detect isolates that were resistant and non-resistant to fluoroquinolones (isolates were assigned to phylogroups as described by Clermont *et al*.^40^). However, their results were for 119 dust samples from pig and poultry barns. To compare the 125 dust samples analysed for fluoroquinolones overall, six further samples from a cattle barn were investigated microbiologically as described by Schulz *et al*.^14^. Information on the detection of *E. coli* in each of the samples is shown in Table S1, which also includes the numbers of barn, sampling periods, animal species housed in the barns, fluoroquinolone concentrations, the presence or absence of *E. coli* isolates in the samples, the phylogroups of the resistant isolates, and the MIC for the non-susceptible isolates. Data on the presence of non-susceptible *E. coli* and the detection of fluoroquinolones in the dust samples were placed into a contingency table (Table 1) for further statistical calculations.

### Statistical analyses

Statistical analyses were performed using SAS 9.3 (SAS Institute Inc., Cary, NC, USA). To investigate the association between the occurrence of non-susceptible *E. coli* and the detection of fluoroquinolones in the dust, the *p*-value from a Fisher exact test and the odds ratio were calculated using the FREQ procedure and data from Table 1. The UNIVARIATE procedure and a signed-rank test were conducted to test the hypothesis that ciprofloxacin, a major metabolite of enrofloxacin, occurs in significantly lower concentrations than the parent drug. We recognized statistical significance when *p* was ≤0.05.

## Supplementary information

Supplementary Information

## Data Availability

All data generated or analysed during this study are included in this published article (and its Supplementary Information files).
