# Supplementary material for: Analysis of fluoroquinolones in dusts from intensive livestock farming and the co-occurrence of fluoroquinolone-resistant Escherichia coli
Source: Sci Rep. 2019 Mar 26;9:5117. doi: 10.1038/s41598-019-41528-z (PMC6435704; doi:10.1038/s41598-019-41528-z)
Supplement: Supplementary file 1 — Supplementary Information [file 41598_2019_41528_MOESM1_ESM.pdf]

Supplementary Information

Analysis of Fluoroquinolones in Dusts from Intensive Livestock Farming and the Co-Occurrence of Fluoroquinolone-Resistant *Escherichia coli*

Jochen Schulz, Nicole Kemper, Joerg Hartung, Franziska Janusch, Siegrun A. I. Mohring and Gerd Hamscher

Supplementary Table S1. Detection of fluoroquinolones and *Escherichia coli* in samples used in this study.

Sample	Sampling Period	Barn	Housed Animals	MFX ng/mg	CIP ng/mg	EFX ng/mg	DiFX ng/mg	*Escherichia coli* growth	Non-susceptible isolates/ phylogroup	MIC values (mg/L)
1-R-S	Dec 2004	2	Pigs	0.07	*	0.11	*	no	no	
2-R-S	Jan 2005	2	Pigs	0.07	*	*	*	yes	yes/B1	> 4
3-R-S	Feb 2005	2	Pigs	1.76	0.07	1.15	*	yes	no	
4-R-S	Mar 2005	2	Pigs	25.57	0.10	0.71	*	yes	no	
5-R-S	Apr 2005	2	Pigs	8.05	*	0.08	*	yes	yes/B1	> 4
6-R-S	May 2005	2	Pigs	1.15	*	*	*	yes	yes/B1	> 4
7-R-S	Jun 2005	2	Pigs	1.46	*	*	*	yes	yes/B1	> 4
8-R-S	Jul 2005	2	Pigs	1.34	*	*	*	yes	no	
9-R-S	Aug 2005	2	Pigs	16.42	*	0.06	*	yes	yes/A	> 4
10-R-S	Sep 2005	2	Pigs	8.89	*	0.08	*	yes	yes/A	> 4
11-R-S	Oct 2005	2	Pigs	0.46	*	0.05	*	no	no	
12-R-S	Nov 2005	2	Pigs	2.54	*	*	*	yes	yes/B1	> 4
13-R-S	Dec 2005	2	Pigs	0.82	0.08	0.74	*	yes	yes/A	> 4
14-E-MS	May 2007	3	Pigs	*	0.03	0.27	*	no	no	
15-E-MS	May 2007	3	Pigs	*	0.03	0.12	*	no	no	
16-E-MS	Mar 2009	3	Pigs	0.23	*	*	*	no	no	

17-E-MS	Mar 2009	3	Pigs	0.15	*	0.02	*	no	no
18-E-MS	Apr 2009	3	Pigs	5.99	*	0.07	*	no	no
19-E-MS	Apr 2009	3	Pigs	0.67	*	0.21	*	no	no
20-E-MS	Apr 2009	3	Pigs	0.22	0.02	0.54	*	no	no
21-E-MS	Apr 2009	3	Pigs	0.09	*	0.05	*	no	no
22-E-MS	May 2009	3	Pigs	0.06	*	0.08	*	no	no
23-E-MS	May 2009	3	Pigs	0.21	*	1.24	*	no	no
24-E-MS	May 2009	3	Pigs	0.02	*	0.06	*	yes	no
25-E-MS	Jun 2009	3	Pigs	0.09	*	0.20	*	no	no
26-E-MS	Jun 2009	3	Pigs	0.04	0.02	0.30	*	yes	no
27-E-MS	Jun 2009	3	Pigs	0.07	0.03	0.38	*	no	no
28-E-MS	Jun 2009	3	Pigs	0.06	*	0.43	*	no	no
29-E-MS	Jun 2009	3	Pigs	0.07	0.03	0.40	*	no	no
30-E-MS	Mar 2009	4	Pigs	0.49	*	*	*	no	no
31-E-MS	May 2009	4	Pigs	0.03	*	0.03	*	no	no
32-E-MS	Jun 2009	4	Pigs	0.05	*	0.02	*	no	no
33-E-MS	Jun 2009	4	Pigs	0.04	*	0.06	*	no	no
34-E-MS	Jun 2009	4	Pigs	0.03	*	0.08	*	no	no
35-E-MS	Jun 2009	4	Pigs	0.03	0.04	0.18	*	no	no
36-E-MS	Jun 2009	4	Pigs	0.02	*	0.12	*	no	no
37-Re-MS (546)	Jan 2000	5	Pigs	*	*	*	*	no	no
38-Re-MS (547)	Jan 2000	5	Pigs	*	*	*	*	no	no
39-Re-MS (548)	May 2000	5	Pigs	*	*	*	*	no	no
40-Re-MS (549)	Mar 2000	5	Pigs	*	*	*	*	no	no
41-CE-MH	Aug 2003	6	Broilers	*	*	*	*	no	no
42-CE-MH	Aug 2003	6	Broilers	*	*	*	*	no	no
43-CE-MH	Sep 2003	6	Broilers	*	*	*	*	no	no

44-CE-MH	Sep 2003	6	Broilers	*	*	*	*	no	no	
45-CE-MH	Nov 2003	6	Broilers	*	*	*	*	no	no	
46-CE-MH	Nov 2003	6	Broilers	*	*	*	*	yes	no	
47-R-MH	Feb 2004	7	Broilers	*	*	0.04	*	yes	yes/B1	> 4
48-R-MH	Feb 2004	7	Broilers	*	*	0.04	*	yes	yes/B1	> 4
49-R-MH	May 2004	7	Broilers	*	*	0.11	*	yes	yes/B1	> 4
50-R-MH	Jun 2004	7	Broilers	*	*	*	*	yes	yes/B1	> 4
51-R-MH	Dec 2004	7	Broilers	*	*	*	*	yes	yes/B1	> 4
52-R-MH	Dec 2004	7	Broilers	*	*	*	*	yes	yes/B1	> 4
53-R-MH	Dec 2004	7	Broilers	*	*	*	*	yes	yes/B1	> 4
54-R-MH	Dec 2004	7	Broilers	*	*	*	*	yes	yes/E	> 4
55-R-MH	Jan 2005	7	Broilers	*	*	*	36.70	yes	yes/D	> 4
56-R-MH	Jan 2005	7	Broilers	*	*	*	41.93	yes	no	
57-R-MH	Jan 2005	7	Broilers	*	*	*	46.35	yes	no	
58-R-MH	Jan 2005	7	Broilers	*	*	*	42.93	yes	no	
59-R-MH	Jan 2005	7	Broilers	*	*	*	21.57	no	no	
60-R-MH	Mar 2005	7	Broilers	*	*	*	0.52	yes	yes/D	> 4
61-R-MH	Mar 2005	7	Broilers	*	*	*	0.33	yes	no	
62-R-MH	Apr 2005	7	Broilers	*	14.94	17.30	0.03	yes	yes/D	> 4
63-R-MH	Apr 2005	7	Broilers	*	15.06	20.43	0.06	yes	yes/B1	> 4
64-R-MH	Jun 2005	7	Broilers	*	*	*	0.02	yes	yes/B1	> 4
65-R-MH	Jul 2005	7	Broilers	*	*	*	*	yes	yes/B1	> 4
66-R-MH	Sep 2005	7	Broilers	*	*	*	*	yes	no	
67-R-MH	Dec 2005	7	Broilers	*	*	*	*	yes	no	
68-R-MH	Dec 2005	7	Broilers	*	*	*	0.02	yes	yes/B1	> 4
69-R-LH	Apr 2005	8	Laying hens	*	*	*	*	yes	no	
70-R-LH	Apr 2005	8	Laying hens	*	*	*	*	yes	no	

71-R-LH	Apr 2005	8	Laying hens	*	*	*	*	yes	no	
72-R-LH	Jun 2005	8	Laying hens	*	*	*	*	yes	no	
73-R-LH	Jun 2005	8	Laying hens	*	*	*	*	yes	no	
74-R-LH	Jun 2005	8	Laying hens	*	*	*	*	yes	no	
75-R-LH	Nov 2005	8	Laying hens	*	*	*	*	yes	no	
76-R-LH	Nov 2005	8	Laying hens	*	*	*	*	yes	no	
77-R-LH	Nov 2005	8	Laying hens	*	*	*	*	yes	no	
78-S-LH	Mar 2009	9	Laying hens	*	*	*	*	no	no	
79-BV-MH	Jun 1994	10	Broilers	*	*	*	*	yes	yes/A	2
80-BV-MH	Jul 1994	10	Broilers	*	*	*	*	yes	yes/A	> 4
81-BV-MH	Aug 1994	10	Broilers	*	*	*	*	yes	no	
82-BV-MH	Sep 1994	10	Broilers	*	*	*	*	yes	no	
83-BV-MH	Oct 1994	10	Broilers	*	*	*	*	no	no	
84-BB-MH	Mar 1992	11	Broilers	*	*	*	*	no	no	
85-BB-MH	Oct 1993	11	Broilers	*	*	*	*	no	no	
86-V-MH	Jun 1992	10	Broilers	*	*	*	*	no	no	
87-V-MH	Jul 1992	10	Broilers	*	*	*	*	no	no	
88-V-MH	Dec 1992	10	Broilers	*	*	*	*	no	no	
89-V-MH	Feb 1994	10	Broilers	*	*	*	*	no	no	
90-V-MH	Oct 1994	10	Broilers	*	*	*	*	no	no	
91-R-R	Feb 2005	14	Cattle	0.01	*	*	*	no	no	
92-R-R	Apr 2005	14	Cattle	0.10	*	*	0.40	no	no	
93-R-R	Apr 2005	14	Cattle	0.02	*	*	*	no	no	
94-R-R	Jun 2005	14	Cattle	0.04	*	*	*	no	no	
95-R-R	Aug 2005	14	Cattle	0.04	*	*	*	no	no	
96-R-R	Aug 2005	14	Cattle	0.02	*	*	*	no	no	
97-R-ME	Sep 2003	12	Ducks	*	*	0.03	*	no	no	

98-R-ME	Mar 2004	12	Ducks	*	*	*	*	yes	no	
99-R-ME	Jan 2005	12	Ducks	*	*	*	*	no	no	
100-R-ME	Nov 2005	12	Ducks	*	*	*	*	no	no	
101-R-P	Jul 2004	13	Turkeys	*	*	*	*	no	no	
102-R-P	Jan 2004	13	Turkeys	*	0.36	0.70	*	yes	yes/B1	> 4
103-R-P	May 2004	13	Turkeys	*	*	0.02	*	yes	yes/B1	> 4
104-R-P	Dec 2004	13	Turkeys	*	*	*	*	yes	yes/B1	> 4
105-P-P	Oct 2004	13	Turkeys	*	*	*	*	yes	no	
1	Jan 1980	1	Pigs	*	*	*	*	no	no	
12	Aug 1981	1	Pigs	*	*	*	*	no	no	
28	Jul 1982	1	Pigs	*	*	*	*	no	no	
79	Aug 1983	1	Pigs	*	*	*	*	no	no	
105	Aug 1984	1	Pigs	*	*	*	*	no	no	
191	Aug 1985	1	Pigs	*	*	*	*	no	no	
251	Aug 1986	1	Pigs	*	*	*	*	no	no	
279	Aug 1987	1	Pigs	*	*	*	*	no	no	
304	Aug 1988	1	Pigs	*	*	*	*	no	no	
325	Jul 1989	1	Pigs	*	*	*	*	no	no	
360	Sep 1990	1	Pigs	*	*	*	*	no	no	
381	Nov 1991	1	Pigs	*	*	*	*	no	no	
410	Aug 1992	1	Pigs	*	*	*	*	no	no	
429	Aug 1993	1	Pigs	*	*	*	*	no	no	
447	May 1994	1	Pigs	*	*	*	*	no	no	
481	May 1995	1	Pigs	*	*	*	*	yes	no	
511	Jul 1996	1	Pigs	*	*	*	*	no	no	
517	Jul 1997	1	Pigs	*	*	*	*	no	no	
532	Jul 1998	1	Pigs	*	*	*	*	no	no	

542	Aug 1999	1	Pigs	*	*	*	*	no	no

CIP, ciprofloxacin; DiFX, difloxacin; EFX, enrofloxacin; MFX, marbofloxacin; MIC, minimal inhibition concentration; * = < LOD (limit of detection);

Supplementary Table S2. Method validation parameters for the detection of fluoroquinolones.

Parameter	Concentration	MFX	CIP	EFX	DiFX
Inter-day Precision (% RSD, n = 30)	0.05 µg/g	5.7	6.4	7.8	7.1
	1 µg/g	8.3	10.5	9.9	11.6
Intra-day Precision (% RSD, n = 10)	0.05 µg/g	3.9–6.3	3.2–10.4	5.7–10.9	3.4–5.7
	1 µg/g	5.3–10.7	3.5–17.5	6.5–9.4	6.0–15.6
Accuracy[a] (% ± SD)	0.05 µg/g	98.2 ± 5.6	73.7 ± 4.7	86.8 ± 6.8	78.4 ± 5.6
	1 µg/g	91.8 ± 7.6	75.7 ± 7.9	87.6 ± 9.9	78.6 ± 11.6
Linearity range (µg/kg)	0.005–1				
Linearity (r^2)		0.9981	0.9992	0.9947	0.9921
Matrix effect (%)	0.05 µg/g	60.3	48.0	45.7	28.5
	1 µg/g	26.6	13.9	14.8	7.8
LOD (µg/kg)		0.005	0.005	0.005	0.005
LOQ (µg/kg)		0.015	0.015	0.015	0.015

CIP, ciprofloxacin; DiFX, difloxacin; EFX, enrofloxacin; LOD, limit of detection; LOQ, limit of quantification; MFX, marbofloxacin; RSD, relative standard deviation; SD, standard deviation

[a]Given as overall process efficiency derived from the recovery data
